# Single-Cell CD4 and CD8 T-Cell Secretome Profiling Reveals Temporal and Niche Differences in Acute Myeloid Leukemia Following Immune Checkpoint Blockade Therapy

**DOI:** 10.1158/2767-9764.CRC-23-0402

**Published:** 2024-03-06

**Authors:** Jessica L. Root, Poonam N. Desai, Christopher Ly, Bofei Wang, Fatima Zahra Jelloul, Jing Zhou, Sean Mackay, Mansour Alfayez, Jairo Matthews, Sherry Pierce, Patrick K. Reville, Naval Daver, Hussein A. Abbas

**Affiliations:** 1 Department of Leukemia, Division of Cancer Medicine, The University of Texas MD Anderson Cancer Center, Houston, Texas.; 2 School of Biomedical Informatics, The University of Texas Health Science Center, Houston, Texas.; 3 Department of Hematopathology, The University of Texas MD Anderson Cancer Center, Houston, Texas.; 4 IsoPlexis Corporation, Branford, Connecticut.

## Abstract

Acute myeloid leukemia (AML) is a heterogeneous malignancy of the blood primarily treated with intensive chemotherapy. The allogeneic T-cell antileukemic activity via donor lymphocyte infusions and stem cell transplantation suggests a potential role for checkpoint blockade therapy in AML. While clinical trials employing these treatments have fallen short of expected results, a deeper exploration into the functional states of T cells in AML could bridge this knowledge gap. In this study, we analyzed the polyfunctional activity of T cells in a cohort of patients with relapsed/refractory (RelRef) AML treated on the clinical trial (ClinicalTrials.gov identifier: NCT02397720) of combination therapy using azacitidine and nivolumab (Aza/Nivo). We utilized the single-cell polyfunctional multiplexed immune assay IsoPlexis to evaluate the CD4 and CD8 T cells in peripheral blood and bone marrow samples collected before and after immunotherapy. This revealed at a pseudobulk level that the CD4 T cells exhibited higher functional activity post-immunotherapy (post-IO), suggesting that CD4-directed therapies may play a role in RelRef AML. Additional single-cell analysis revealed significant differences in baseline polyfunctionality in bone marrows of responders as compared with nonresponders for both CD4 and CD8 T cells. Overall, this study highlights the impact of polyfunctional assessment in understanding CD4 and CD8 dynamics in contexts of therapy in AML.

**Significance:**

We found T-cell polyfunctionality differs between local and systemic microenvironments. Enhanced variability in proteomic profiles of bone marrow CD4 T cells post-IO suggests their pivotal role in AML treatment response. Single-cell analysis identified novel CD4 and CD8 T-cell functional groups linked to immunotherapy response within the bone marrow.

## Introduction

Acute myeloid leukemia (AML) is a hematologic malignancy characterized by the rapid proliferation of abnormal myeloid cells in the bone marrow (BM; ref. 1). Despite advances in our understanding of the molecular and genetic basis of AML, the prognosis for patients remains poor, with a 5-year survival rate around 25%–30% (2). Recent advancements in cancer immunotherapy, particularly immune checkpoint blockade (ICB) targeting PD-1 or CTLA-4, have revolutionized the treatment landscape for various solid tumors (3–6) via unleashing the cytotoxic potential of T cells (7). However, ICB therapies in AML have not yielded similarly promising results (8–11). In a study assessing the efficacy of ipilimumab, an immune checkpoint inhibitor targeting CTLA-4, in patients who experienced relapse following allogeneic-hematopoietic stem cell transplantation (allo-HSCT), 62% of patients showed no response, while those who achieved complete (CR) or partial response all had some prior GVHD (10), suggesting inherent allogeneic T-cell antileukemic activity. In addition, combining PD-1 inhibitors nivolumab or pembrolizumab with hypomethylating agents in patients with relapsed/refractory (RelRef) AML yielded overall response rates of 33% and 38%, respectively, with only a few cases having long-term remissions (8, 11). Finally, a preclinical melanoma mouse model revealed multiple combinations of low-dose azacitidine (Aza), a hypomethylating agent (HMA), significantly enhanced tumor responses to anti-CTLA-4 ICB therapy, thus supporting the potential of DNA methyltransferase inhibitors, like Aza, to potentiate the antitumor effects of immune checkpoint inhibitors in solid cancers (12). A deeper understanding of T-cell functionality in AML in the context of ICB is essential to determine the underlying reasons for its limited success and to improve the therapeutic potential of immune checkpoint inhibition in leukemia.

Our group and others employed single-cell RNA (scRNA) profiling and demonstrated that responses to ICB in AML are associated with dynamic cellular phenotypes and repertoire expansions (13). Furthermore, we identified distinct CD8 cellular states characterized by terminal differentiation and hyperexpansion in patients with RelRef AML as compared with newly diagnosed patients (14). Additional investigations reveal that cytokine-producing CD8 T cells from patients with AML exhibit both exhausted and senescent features with coexisting phenotypic signatures (15, 16). These senescent-like CD8 T cells demonstrated impaired functionality and were negatively correlated with patient survival (17). The cytolytic activity and infiltration of CD8 and natural killer cells is affected by the specific AML subtype, further complicating our understanding of T-cell functionality (18). To mend this gap, our group performed a proof-of-concept single-cell polyfunctional study on a subset of patients with RelRef AML treated with azacitidine combined with nivolumab (Aza/Nivo) and identified that the polyfunctional activity of CD4 T cells predicted responses to therapy (19). The polyfunctional activity of T cells was measured using the IsoPlexis single-cell polyfunctional multiplex assay which profiled CD4 and CD8 T-cell secretomes. The mean fluorescence intensity (MFI) of the secreted proteins was then used to calculate a polyfunctional strength index (PSI; Materials and Methods). This technology has demonstrated promise in studies evaluating the efficacy of other immune-based therapies such as chimeric antigen receptor (CAR)-T cells (20).

While the aforementioned studies provided insights into T-cell biology in AML, several questions remain to be addressed. First, are T cells functionally similar in the peripheral blood (PB) and BM where leukemic cells reside and circulate, respectively? This question is particularly relevant, as AML cells can create an immune-suppressive microenvironment in the BM, whereas PB T cells may not be as affected (21). Second, how do the polyfunctional profiles change following immune checkpoint therapy in AML? Furthermore, evaluating these aspects at the single-cell level can provide insightful information about the heterogeneity of functional CD4 and CD8 cellular states in AML.

In this study, we leveraged a cohort of adult patent with RelRef AML treated with Aza/Nivo enrolled on a clinical trial (NCT02397720; ref. 22). PB and BM samples were collected from a total of 21 patients prior to and 2 months after immunotherapy (post-IO). CD4 and CD8 T cells were then isolated and the IsoPlexis-based polyfunctional multiplex assay was performed on each cellular subset to thoroughly characterize the polyfunctional profiles of T cells. Functional data were also integrated with clinical and demographic characteristics of these patients. This comprehensive approach allowed us to explore the temporal (pre/post-IO) and niche (PB vs. BM) differences in T-cell functional states in a unique cohort of patients with AML providing insights on T-cell functional states in AML at the single-cell level and in the context of PD-1 blockade therapy.

## Materials and Methods

### Patients

Adult patients (≥18 years old) with AML who had failed prior therapy (included HMA-based therapy) were eligible for this study. Some patient data, including detailed inclusion/exclusion criteria, were reported previously (22). Response to therapy was defined as patients who achieved a CR, a CR with incomplete blood count recovery, a partial response, or hematologic improvement. The study was conducted in accordance with the Declaration of Helsinki (ClinicalTrials.gov identifier: NCT02397720) and MD Anderson Institutional Review Board approval, with written informed consent from all participants.

### Single-cell 32-Plex Functional Proteomic Profiling

Cryopreserved PB and BM samples were thawed and cultured in complete RPMI medium (Thermo Fisher Scientific) supplemented with 10 ng/mL IL2 (BioLegend) at a density of 1 × 10^6^ cells/mL in a 37°C, 5% CO_2_ incubator overnight. After overnight recovery, viable T cells were purified from dead cells using Ficoll-Paque Plus medium (GE Healthcare). CD8^+^ then CD4^+^ T-cell subsets were sequentially enriched using anti-CD8 or anti-CD4 MicroBeads (Miltenyi Biotec), respectively, and resuspended in complete RPMI media at a density of 1 × 10^6^/mL. An aliquot of 100 µL of CD4^+^ or CD8^+^ T-cell suspension was seeded into a well of 96-well flat-bottom plate precoated with anti-human CD3 (clone OKT3, Thermo Fisher Scientific/Invitrogen, 10 µg/mL in PBS at 4°C, O/N) with a supplement of soluble anti-human CD28 (clone CD28.2, Thermo Fisher Scientific/Invitrogen) at a final concentration of 5 µg/mL. After culture at 37°C, 5% CO_2_ for 24 hours, the cells were stained with Alexa Fluor 647-conjugated anti-CD4 or CD8 antibody (BioLegend) at room temperature for 10 minutes, rinsed once with complete RPMI and then resuspended in complete RPMI medium at a density of 1 × 10^6^/mL. A total of 30 µL of CD4^+^ or CD8^+^ T-cell suspension was loaded into an IsoCode Chip (IsoPlexis) and incubated at 37°C, 5% CO_2_ for additional 16 hours. Following this final incubation, subsequently secreted proteins from approximately 1,000 single T cells were captured by the 32-plex antibody barcoded chip and analyzed by backend fluorescence ELISA-based assay. Polyfunctionality of T cells defined as a cell cosecreting two or more cytokines were analyzed by the IsoSpeak software across the five functional groups. The PSI of T cells was computed using a prespecified formula, defined as the percentage of polyfunctional cells, multiplied by the sum of the MFI of the proteins secreted by those cells (20, 23–28). To quantify the polyfunctionality, we used the following cytokine groupings as defined by IsoPlexis: chemoattractive (CCL-11, IP-20, MIP-1β, RANTES), effector (granzyme B, IFNg, MIP-1a, Perforin, TNFa, TNFβ), inflammatory (IL1β, IL6, IL17A, IL17F, MCP-1, MCP-4), stimulatory (GMCSF, IL2, IL5, IL7, IL8, IL9, IL12, IL15, IL21), and regulatory (IL4, IL10, IL13, IL22, TGFβ1, sCD137, sCD40L).

### Single-cell Analysis

Single-cell data and associated metadata were imported into a Seurat object for downstream processing (29). Of the 111,420 single cells, we selected 101,120 by filtering out those cells with over 3,000 MFI to remove outlier values that would skew the analysis, as per standard single-cell analysis methodology. The PSI data were converted into a Seurat object and log normalized via the Seurat function NormalizeData followed by identification of highly variable features. The harmony R package was used to correct for potential batch effects between individual patients (30). Dimensional reduction was performed using principal component analysis and uniform manifold approximation and projection (UMAP).

Differential abundance analysis was carried out utilizing the miloR R package (version 1.6). Utilizing a harmonized dimensional reduction approach, a k-nearest neighbor graph was generated encompassing partially overlapping neighborhoods. Differential abundance across these neighborhoods was assessed considering the timepoints (pre- vs. post-IO) as variables of interest. A FDR procedure was implemented to account for multiple hypothesis testing.

### Data Visualization

The ComplexHeatmap and pheatmap R packages were used to construct heat maps. The ggpubr package was used for boxplot creation. The gtsummary package was used for table creation.

### Statistical Analysis

Statistical analyses were performed using base R (version 4.2.2.) and the rstatix package. Bulk data used pairwise *t* tests. Single-cell data statistics were calculated using the paired Wilcoxon rank-sum test. A FDR of *q* less than 0.05 was used for multiple hypothesis testing. *P* values less than 0.05 were considered significant.

### Data Availability Statement

The data generated in this study and all related codes are available at the following Github repository: https://github.com/abbaslab/2023_AML_Tcell_Secretome_Isoplexis.

## Results

### Patient Characteristics and Treatments

**FIGURE 1:**
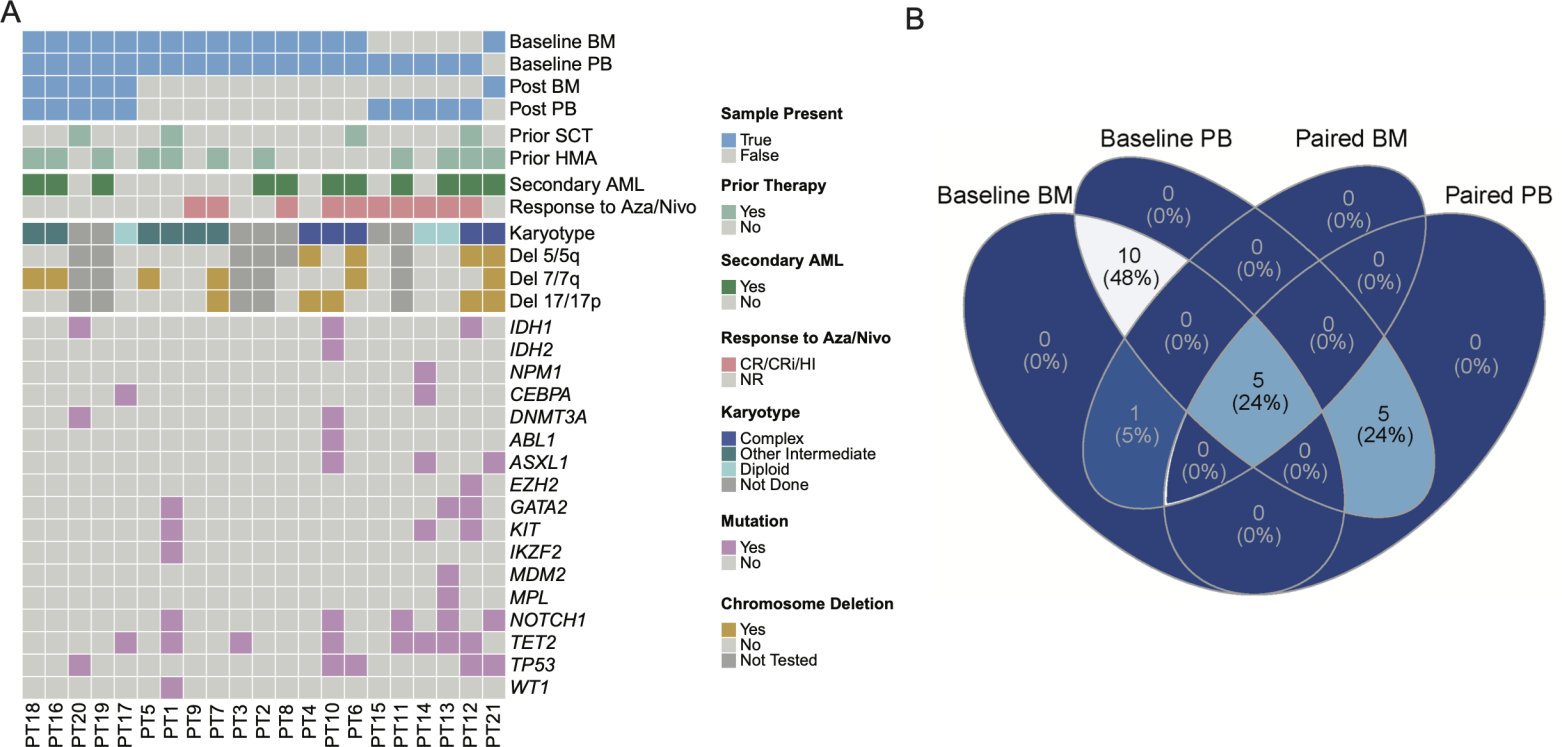
Integrative analysis approach and clinical characteristic profile of patient cohort. **A,** Clinical characteristics of RelRef AML cohort for 21 patients involved in the phase II clinical trial. Among the 21 patients enrolled, 20 had baseline PB samples, 16 had baseline BM samples, 10 had posttreatment PB samples, and 6 had posttreatment BM samples. Four patients had prior allogeneic stem cell transplantation (all-SCT) therapy; 11 patients had prior HMA therapy; 11 patients had Secondary AML; 10 patients had CR to Aza/Nivo immunotherapy in the phase II clinical trial; Karyotype and molecular diagnostics at baseline for all 21 patients are included. **B,** Venn diagram visualizing the patient cohort.

To investigate differences in T-cell polyfunctionality, we investigated the secreted proteome of activated bead-sorted CD4 and CD8 T cells using the IsoPlexis 32-plex human adaptive immune panel from 21 patients with RelRef AML enrolled in a phase II clinical trial using combination Aza/Nivo therapy [10 patients were responders and 11 patients were nonresponders (NR)] (Fig. 1A). Briefly, the median patient age at time of study enrollment was 68 years (range, 47–90) with 71% being male, while the median time to response was 3.38 months (range, 0.8–12.6; Supplementary Table S1).

Of the 21 patients with AML evaluated in this study, pretreatment PB and BM specimens were obtained from 20 and 16 patients, respectively, serving as baseline measurements for the analysis. Among the 16 patients with baseline BM samples, 15 patients also had baseline PB samples. After treatment with Aza/Nivo, PB samples from 10 patients and BM samples from 6 patients were evaluated allowing for a longitudinal polyfunctional T-cell analysis following ICB therapy (Fig. 1B). To assess differences in cytokine dynamics among T-cell subsets, we isolated CD4 and CD8 T cells from each sample using microbead enrichment, followed by CD3/CD28 T-cell stimulation, and measurement of secreted proteins. Polyfunctional groups were assigned on the basis of the IsoPlexis standard recommendation, including chemoattractive, effector, inflammatory, regulatory, and stimulatory functional groups (see Materials and Methods), allowing for analysis and comparison of different functional characteristics of T cells in the local and systemic environments. In total, we characterized CD4 and CD8 T cells from 52 patient samples, including 30 PB and 22 BM samples, from 21 patients, which included PB-BM pairs and longitudinal samples, constituting a substantial resource for T-cell functional in AML.

### Establishing Baseline Functionality for T Cells in AML

**FIGURE 2:**
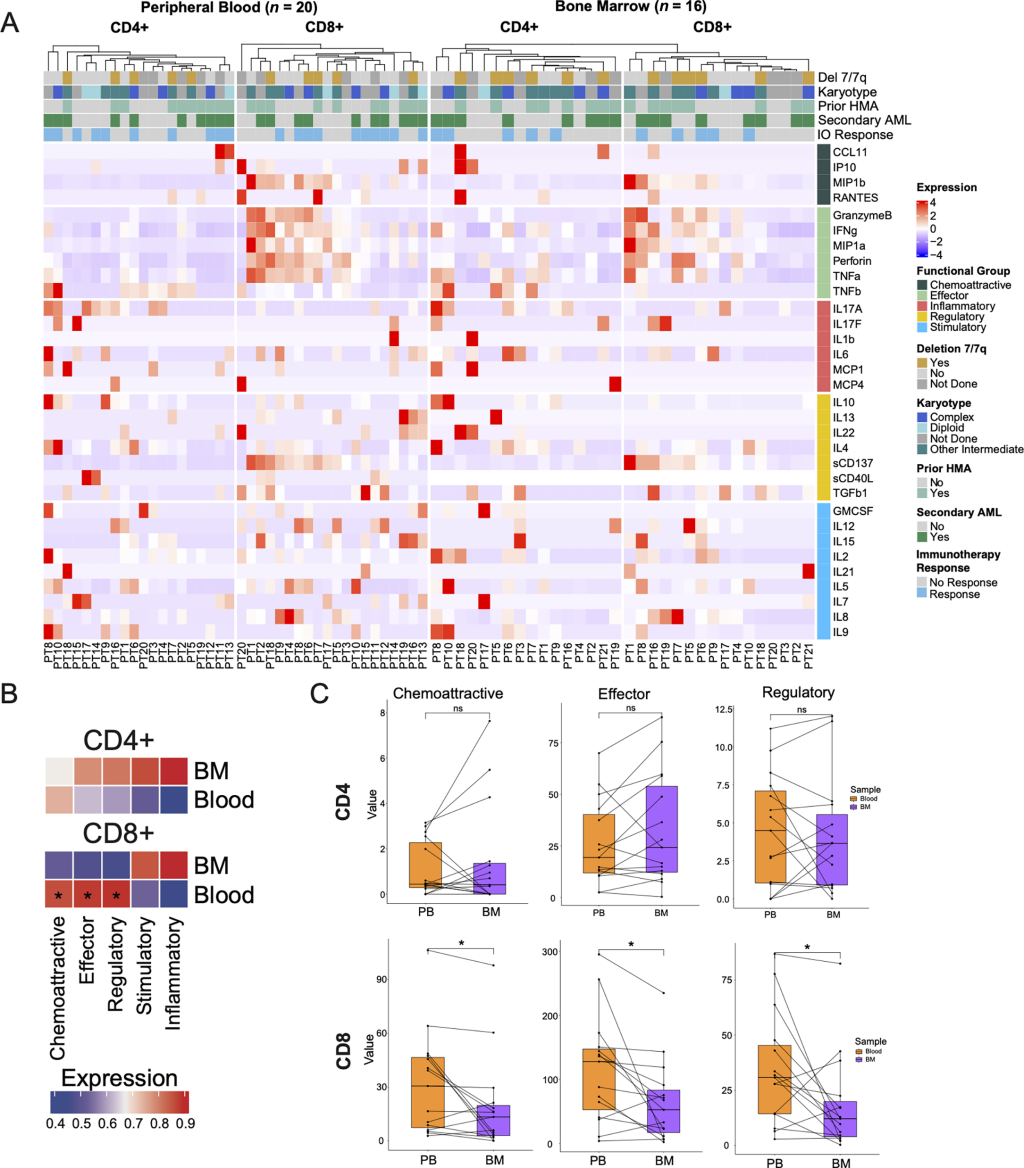
Baseline T-cell functional activity in PB and BM. **A,** Heat map of T-cell cytokine expression values in PB (*n* = 20) and BM (*n* = 16) samples at baseline split by cell subset and scaled by row with clinical characteristic and functional group annotations. ComplexHeatmap was used for data analysis and visualization. **B,** ComplexHeatmap comparing cytokine expression based on functional groups of CD4 and CD8 cells between TMEs at baseline in the PB and BM. **C,** Bar plot representation of the significant functional groups from B. Pairwise analysis using ggpaired shows differences based on functional group expression compared between TMEs (*P* < 0.05).

The functional proteomic data from the 20 PB and 16 BM baseline samples were explored at the pseudobulk level to evaluate activity of the 32 individual cytokines from the five polyfunctional groups in CD4 and CD8 T cells (Supplementary Fig. S1A). The PSI is defined as the product of the polyfunctionality of single T cells (the percentage of profiled single cells secreting two or more cytokines) and the intensity of those cytokines secreted by the polyfunctional single cells (Materials and Methods). Elevated PSI values correspond to increased secretion of multiple cytokines per individual cell. Exploratory analysis revealed similar patterns of CD8 effector cytokine expression in both the BM and PB at baseline (Fig. 2A). Further analysis highlighted differences in T-cell baseline expression based on polyfunctional groups and individual cytokines. The chemoattractive, effector, and regulatory groups exhibited higher baseline activity from CD8 T cells in PB (Fig. 2B and C). Individual cytokines, including granzyme B, IFNγ, IL15, IL17A, MIP-1α, MIP-1β, Perforin, sCD137, and TNFα had significantly higher baseline expression in CD8 compared with CD4 T cells (Supplementary Fig. S1B). Similarly, polyfunctional activity in the BM was higher for those groups, though fewer patients showed increased chemoattractive and regulatory activity (Supplementary Fig. S1C). Finally, the major difference in activity between CD4 and CD8 in the regulatory group appears to be driven by sCD137 (Fig. 2A; Supplementary Fig. S1B). This individual cytokine is important for CD8 T lymphocytes undergoing antigen activation, as the CD137 ligation with cognate ligand or agonist antibodies leads to protection from apoptosis and promotes proliferation and cytokine secretion (31, 32). Thus, the more sCD137 is secreted from CD8 T cells, the higher the potential for its polyfunctional activity. These findings are consistent with CD8 T cells having a higher cytokine polyfunctionality than CD4 T cells prior to treatment.

Further baseline exploratory analysis for age demonstrated positive correlations with sCD40 L from PB CD4 T cells, and GMCSF and IL7 from CD4 T cells in the BM. Conversely, negative correlations with age were observed for IL2 from CD4 T cells in the blood and MIP1b from CD8 T cells in the BM. Notably, the only age-related correlation with the functional groups involved a negative association with the chemoattractive group from CD8 T cells in the BM (Supplementary Fig. S2). This corroborates previously found information that older patients secrete less IL2 and have memory cell inflation (33).

### CD8 Polyfunctionality Differs in PB and BM Compartments

To determine whether local (BM) T cells are functionally distinct from systemic (PB) T cells, we compared the baseline polyfunctional activity of the 10 patients with paired PB and BM specimens that were collected simultaneously prior to therapy initiation (Supplementary Fig. S3A). Except for IL8 expression that was significantly higher in CD4 T cells in the BM as compared with the PB (*P* = 0.00867; Supplementary Fig. S3B), there were no significant differences observed in the polyfunctional groups from CD4 T cells when comparing PB and BM (Fig. 2C). Of note, IL8 is a chemoattractant for neutrophils and has been implicated in microenvironment remodeling in solid tumors (34). In CD8 T cells, chemoattractive, effector, and regulatory polyfunctional groups exhibited significantly higher expression in PB than in BM (Fig. 2C). Individually significant cytokine profiles that were different in CD8 T cells include granzyme B (*P* = 0.043), IL4 (*P* = 0.013), MIP-1β (*P* = 0.027), sCD137 (*P* = 0.037), TNFα (*P* = 0.00486), and TNFβ (*P* = 0.045; Supplementary Fig. S3C). These findings indicate that the BM microenvironment significantly impacts CD8 T-cell functionality likely due to the local effect of AML cells on CD8 T cells. This effect may be dampened in the PB where the direct AML-T cell contact may be less conspicuous.

### CD4 T Cells have Differential Effector Cytokine Activity Between Responders and Nonresponders

Pairwise analysis of baseline and 2-month post-IO samples revealed similar response patterns in CD4 and CD8 T cells in both PB (*n* = 10) and BM (*n* = 6) samples. CD4 T cells in PB showed significant increase in the expression of chemoattractive (*P* = 0.029), effector (*P* = 0.049), and regulatory (*P* = 0.020) cytokines with BM CD4 T cells trending similarly (Supplementary Fig. S4A). Significant individual cytokine profile changes in CD4 T cells were also observed (Supplementary Fig. S4B and S4C). CD8 polyfunctionality post-IO revealed no significant differences in polyfunctional groups or individual cytokine profiles (Supplementary Fig. S4D). While this demonstrates the immunotherapy affects T cell functional activity, it does not account for response status.

**FIGURE 3:**
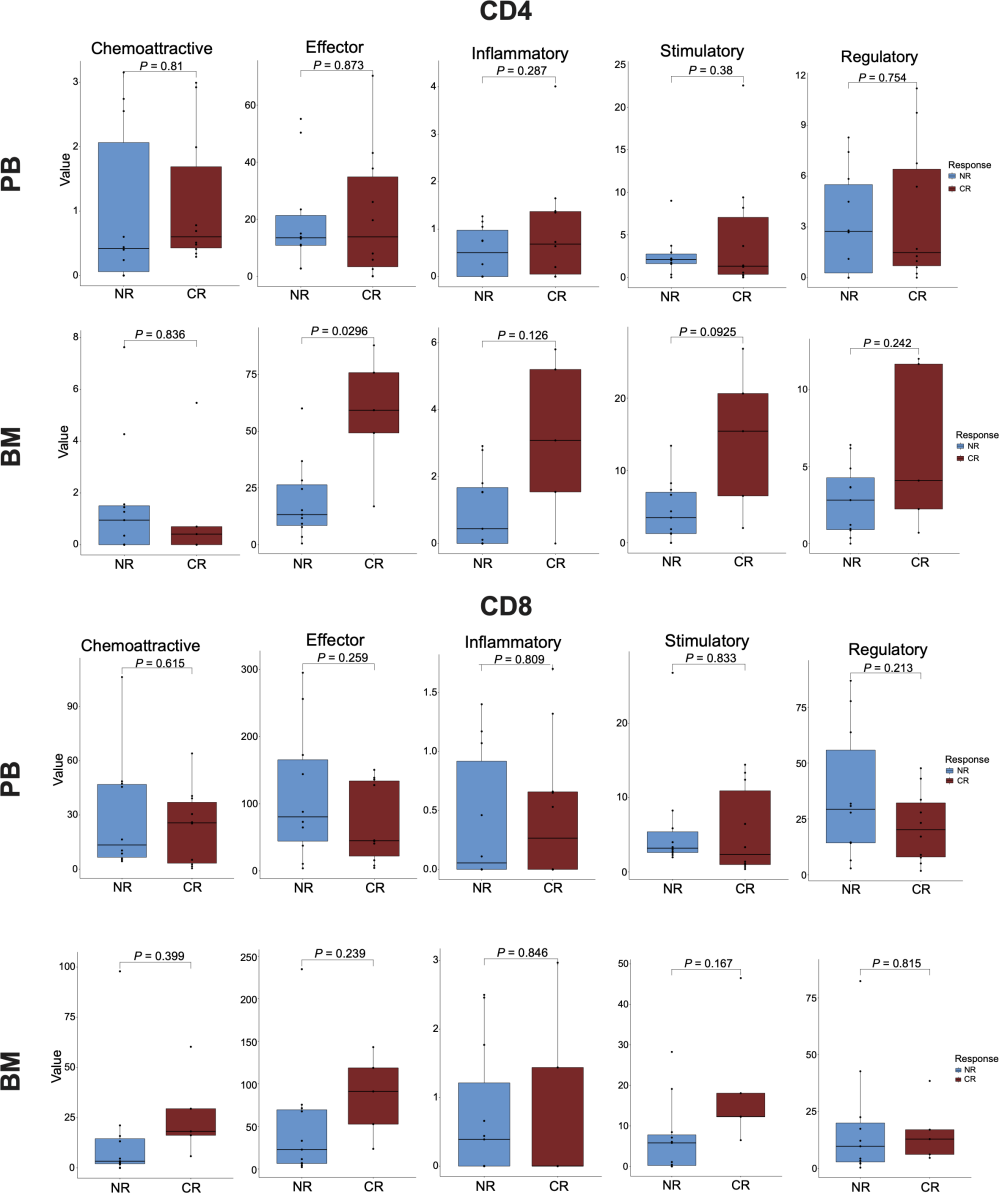
Exploratory analysis of CR and NR patients at baseline. CRs have significantly higher CD4 effector cytokine expression than NRs in the BM. No significant difference in functional group expression from CD8 cells in either PB and BM between CR and NR.

Interestingly, comparisons between CR and NR patients prior to immunotherapy did not yield significant differences in polyfunctionality in CD8 T cells, but there was a significant increase in CD4 BM effector cytokine functional activity in CR (Fig. 3). Given that there was no observable difference in effector cytokine functionality in the PB, this indicates a potential distinction in the local versus systemic tumor microenvironment (TME) effect. Recent studies suggest CD4 T cells are critical for antitumor responses (35, 36). In addition, effector CD4 T cells in RelRef AML were associated with better outcomes and responses (19), and tumor-infiltrating CD4 T cells were found to exhibit T helper and cytotoxic activity (37). These findings suggest CD4 T cells may play a significant role in inducing response to immunotherapy in AML.

### Single-cell Analysis Identifies Similarities Between Pre- and Post-IO T Cells

**FIGURE 4:**
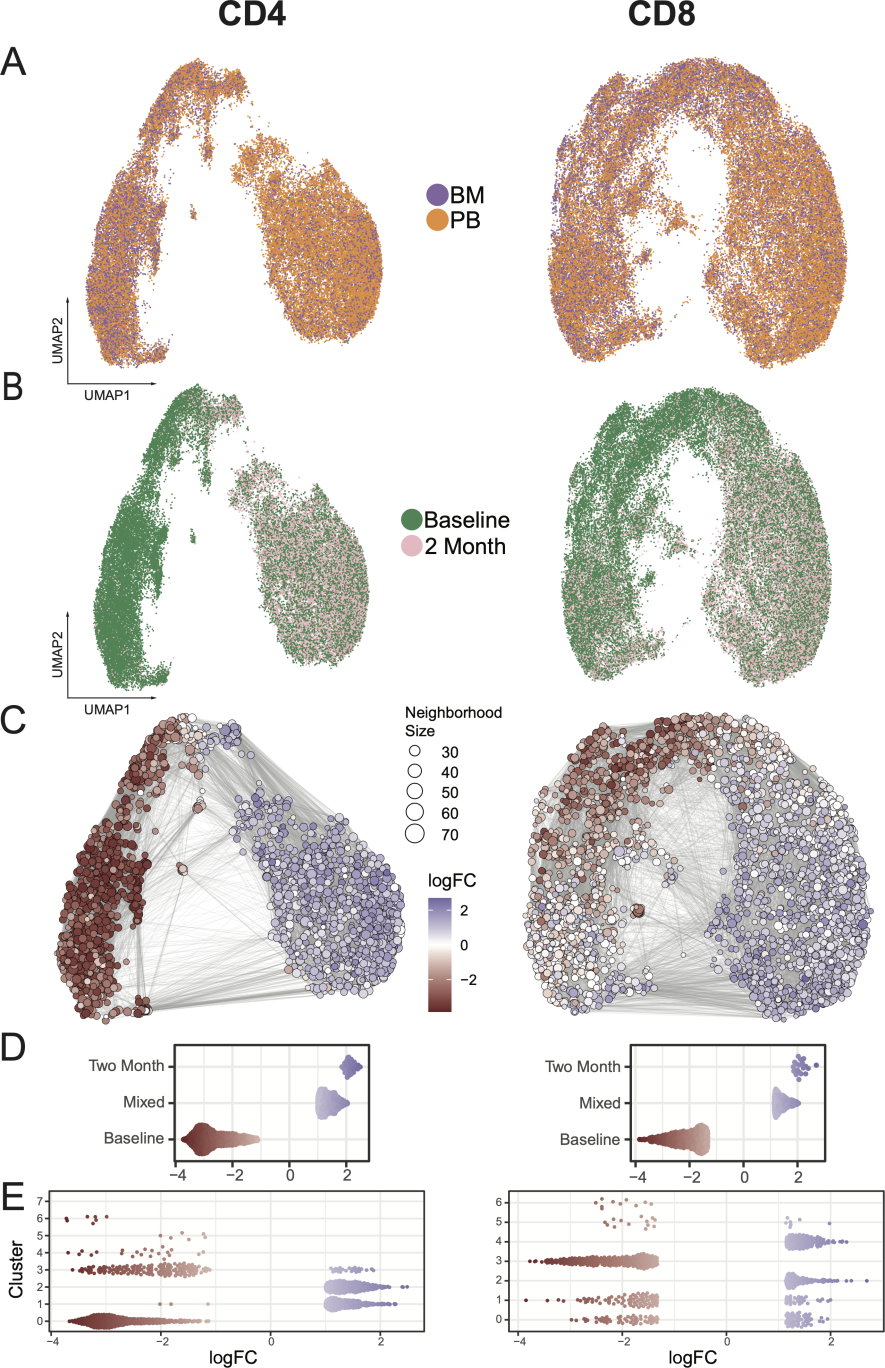
Single-cell analysis of IsoPlexis data for CD4 cells (left) and CD8 cells (right). **A,** UMAP visualization colored by sample location (orange for PB, purple for BM). **B,** UMAP colored by time point (green for baseline, pink for post-therapy). **C,** Neighborhood graph depicting differential abundance testing results obtained from miloR. Colors represent the log fold-change between baseline (red) and post-IO (blue) cells. White neighborhoods are nondifferential (FDR 10%). The edges depict links between cells shared by neighborhoods. **D,** Beeswarm plot of the distribution of neighborhoods by timepoint. **E,** Beeswarm plot of the distribution of neighborhoods by UMAP-based cluster.

To further explore T-cell cytokine responses to immunotherapy, we expanded upon the initial pseudobulk approach by processing the single-cell functional proteomic data using a bioinformatics pipeline adapted from the standard Seurat R package scRNA sequencing (scRNA-seq) approach (29). This approach allows us to fully leverage the power of the single-cell polyfunctional analysis. A total of 101,120 cells successfully passed quality control checks (see Materials and Methods; Supplementary Fig. S5). Utilizing the resulting sample-level batched correlation UMAP projection, eight distinct CD4 and seven distinct CD8 clusters were identified, with BM and PB cells dispersed across all clusters (Fig. 4A; Supplementary Fig. S5). Notably, there were variations in cell clustering based on the time of therapy, as some clusters exclusively comprised cells with baseline values, while others exhibited a mixture of baseline and 2 months post-IO values (Fig. 4B). This suggests the dynamic functional states of CD4 and CD8 T cells in AML following ICB-based therapy.

Given the overlap between baseline and post-IO T cells on the UMAPs (Fig. 4B), we investigated whether there were any unique aspects of post-IO cells. Neighborhoods of similar cells, based on differences in abundance between cells at baseline and 2 months post-IO, were determined utilizing a cluster-independent generalized linear model framework, implemented through the miloR package (38). This framework facilitated the calculation of log fold-change values between samples from the distinct timepoints within each identified cellular neighborhood (Fig. 4C). To further understand the neighborhood-level differences between the timepoints, we utilized beeswarm plots to clearly show how the neighborhoods are distributed across the timepoints and clusters. In CD4 T cells, miloR identified 2,158 neighborhoods, of which 982 (45.5%) were composed only of baseline cells and 29 (1.3%) were composed only of post-IO cells (FDR < 0.1; Fig. 4D, left). In CD8 T cells, miloR identified 2,706 neighborhoods, of which 1,097 (40.5%) were composed only of baseline cells and 23 (0.85%) were composed only of post-IO cells (FDR < 0.1; Fig. 4D, right). Upon examining the cluster-based separation of neighborhoods for CD4 T cells, a distinct bias was observed in each cluster, with clusters one and two primarily containing mixed or 2-month neighborhoods (Fig. 4E, left). The CD8 analysis revealed separation of baseline and mixed/2-month neighborhoods in clusters 0 and 1, highlighting localized groupings of baseline cells exhibiting a mixed phenotype (Fig. 4E, right). This indicates that the post-IO compartment does not represent a distinct functional state, as pre- and post-IO cells could not be separated through neighborhood analysis. This finding suggests that patient responses to immunotherapy may be predetermined prior to treatment.

### Single-cell Analysis Reveals Differences Between Responders and Nonresponders

**FIGURE 5:**
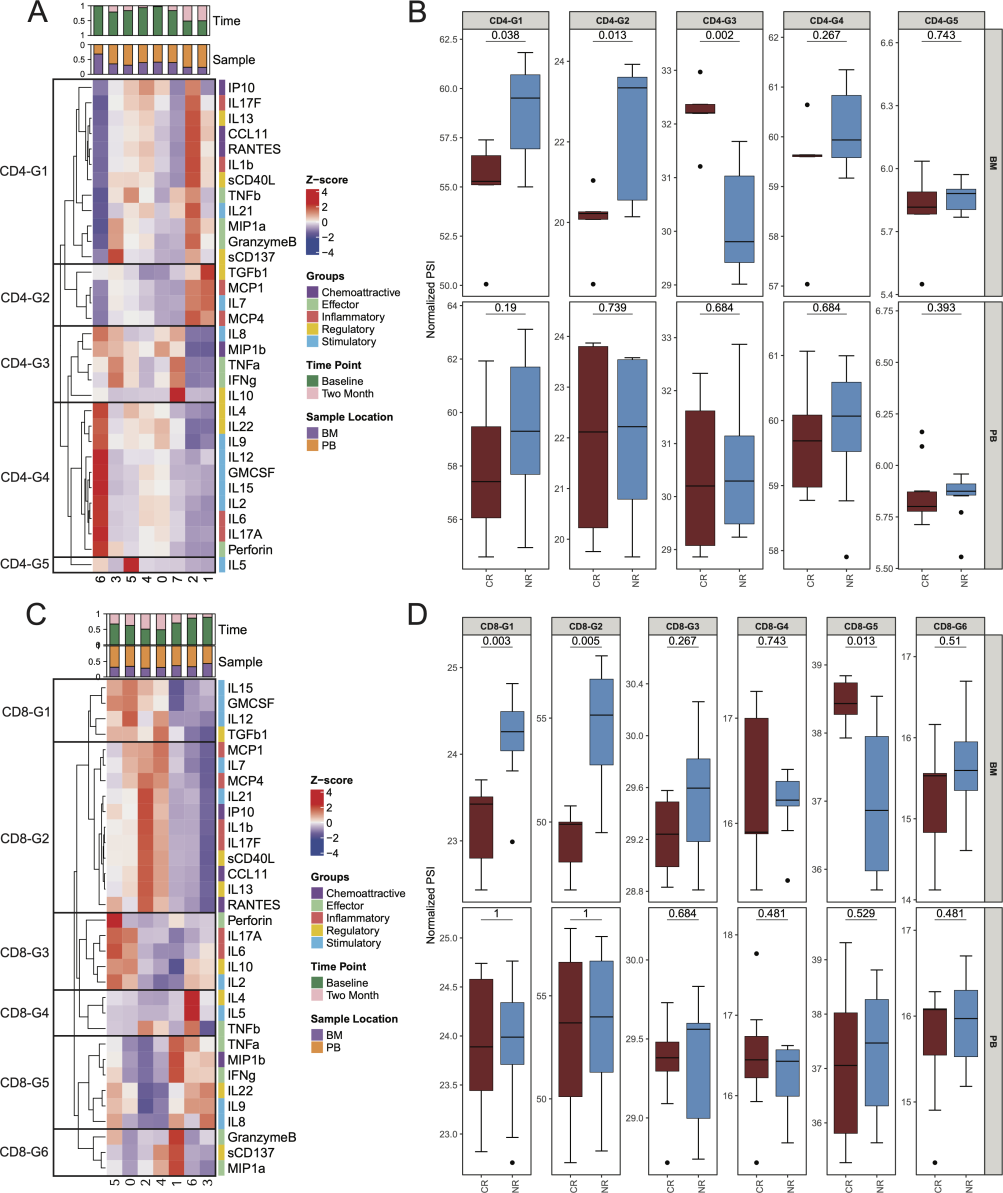
Single-cell cytokine and polyfunctional group analysis at baseline. Heat map of cytokine expression by cluster for **A**. CD4 T cells. Annotation bars show cluster breakdown by sample location and timepoint. Hierarchical clustering was used to create new polyfunctional groups (CD4-G1-5), which are highlighted by black boxes. **B,** Boxplots for CD4 T cells show the comparison of complete responders (CR, brown) and nonresponders (NR, blue) at baseline by sample location in these newly defined polyfunctional groups for T cells. **C,** Heat map of cytokine expression by cluster for CD8 T cells with hierarchical clustering creating new polyfunctional groups (CD8-G1-6). **D,** Boxplots for CD8 T cells show comparison of CR (brown) and NR (blue) at baseline.

A detailed look at polyfunctionality at the single-cell level indicates that clusters were not delineated by the predefined polyfunctional groups (Fig. 5A–C). We therefore leveraged the unbiased, unsupervised clustering of T-cell functionality to reveal novel functional groups at a single-cell level at baseline. Using this approach, we identified five distinct functional groups for CD4 and six for CD8. None of these functional groups, either for CD4 or CD8, demonstrated any differential relationship with response in the T cells from the PB (Fig. 5B and D). However, these novel T-cell functional groups were correlated with responses in microenvironmental T cells from the BM. In particular, a higher proportion of CD4 and CD8 T cells with cosecretion of IFNγ, TNFα, MIP-1β, and IL8 from the BM, CD4-G3, and CD8-G5 were associated with CR compared with NR (*P* = 0.002 for CD4-G3 and *P* = 0.013 for CD8-G5, Fig. 5B and D). This corresponds with data previously reported demonstrating the importance of IFNγ and TNFα in response to immunotherapy (39, 40). This highlights the importance of effector CD4 and CD8 T cells in response to immunotherapy in the local immune microenvironment, which may not be fully reflected in the PB of these patients. In addition, using an unbiased single-cell approach, we were able to further disentangle the complex T-cell functionality induced by immunotherapy and correlating with disease response that was not fully appreciated at the pseudobulk level.

## Discussion

Although immunotherapy has demonstrated limited efficacy in AML, it has seen clinical success in improving outcomes for other malignancies. Allogeneic-HSCT and donor lymphocyte infusions have shown improved outcomes in patients with AML through the antileukemic activity of donor T cells (41). In addition, immunotherapies offer significant advantages over standard chemotherapies through accommodating tumor heterogeneity and leveraging the patient's individual immune repertoire. Indeed, the use of HMAs can enhance underlying mechanisms of the antitumor immune response (42). A study in patients with myelodysplastic syndrome examined global T-cell receptor (TCR) repertoires comparing clonotypes in responders and NR to HMA. Results revealed novel clonotypes in responders, suggesting potential recruitment of T cells from systemic tissues or response from BM-residing T cells. However, clonotype contraction was observed in NR following HMA treatment. The distinct clonotype dynamics are likely attributed to HMA therapy, though the direct effects of HMA on T cells remains unclear (43). Thus, there is potential for leveraging T cells as a therapeutic strategy in combating hematologic malignancies, including AML, but our current understanding does not afford for us to effectively do so.

Utilization of single-cell polyfunctional assays to assess the immune response to treatment is paramount in broadening our understanding of T-cell functionality. Proteomic profiling has been established as a predictive tool for patient responses to AML, and CD4 polyfunctionality has been identified as a marker for response to immunotherapy (19, 44). Indeed, CD19 CAR-T cells collected from patients with chronic lymphocytic leukemia who responded to therapy were not only polyfunctional, but persisted and maintained their polyfunctionality up to 4 years postinfusion (45).

We assessed the polyfunctionality of CD4 and CD8 T cells in PB and BM at relapse and post-IO in patients with RelRef AML which revealed similar response patterns in CD4 and CD8 T cells in both PB and BM samples. Recent studies suggest CD4 T cells are critical for antitumor responses (35, 36). Our analysis suggests that PB CD4 T cells could potentially serve as an indicator of T-cell functional activity within the BM of RelRef AML at a pseudobulk level, avoiding the need for invasive BM extraction procedures. Further assessment of T cells in the BM TME may have important implications for therapeutic decisions. For example, T cells play a critical role in post-chemotherapy immune reconstitution and oral-AZA maintenance therapy. Immune profiling was performed on the BM of patients with AML at remission and on-treatment to identify potential prognostic immune features. Results found that oral-AZA modulates T-cell activity in the BM TME by maintaining the setting of AML after chemotherapy (46). Though these immune-mediated responses could be associated with clinical outcomes based on the study, it still requires sampling the BM as T-cell functional similarities between PB and BM remains unclear.

One study comparing the T-cell functions in the PB versus BM TMEs of patients with AML found T-cell suppression in both TMEs, but did not reveal significant or consistent functional differences between TME resident T cells. To further understand the T-cell suppression mechanism, blocking antibodies were added against checkpoint molecules PD-1, CTLA-4, and TIM3 to proliferation assays, which revealed PD-1 blockade as being the most effective at inducing T-cell proliferation in 67% of the initial nonproliferating groups (47). Though such findings do suggest the T-cell suppressive effect in AML is complex and likely involves multiple mechanisms, the T-cell functional differences between TMEs in AML are still unclear. Further analysis is warranted to ascertain the degree of the similarity and potential therapeutic implications.

Examining the temporal impact of cytokine dynamics reveals that CD4 T cells exhibited greater variation in their cytokine profiles post-IO. The observed increase in functionality could be attributed to their lower baseline function, thus enabling a more pronounced response to PD-1 blockade therapy. *In vitro* studies demonstrated PD-1 blockade partially reversed CD8 dysfunction (15), but no significant differences in cytokine secretion or polyfunctionality were observed in CD8 cells of patients with AML post-IO. This suggests that CD4-directed therapy may be more effective than CD8-directed therapy at the time of relapse.

We also note how bulk and single-cell analyses synergized to provide a more comprehensive understanding of polyfunctional responses in T cells. Drilling down into the single-cell data determined that CD8 T cells were more affected by the outlier values that were removed in the single-cell approach and masked the polyfunctional differences between response groupings. Single-cell clustering demonstrated that post-IO T cells were not uniquely defined, but shared properties with baseline T cells. A similar result was seen in our previous analysis of scRNA-seq data of CD8 T cells where there was a gradient of differentiation and potential T-cell dysfunction that was defined prior to treatment (14).

Limitations of this study include the sample availability from the patient cohort, as all patients with posttreatment BM samples were status NR. In addition, the study did not consider the potential impact of secondary AML or previous treatment regimens on the polyfunctional states of T cells due to the small sample size. Furthermore, there is a lack of established methods for analyzing cytokine data in the single-cell analysis, and single-cell RNA-based approaches may not fully capture the complexity of cytokine profiles. The IsoPlexis technology, while innovative, has its limitations. Stimulating CD4 and CD8 T cells outside their native microenvironment may not capture the full spectrum of their *in vivo* responses. The cytokines measured by IsoPlexis offer only a partial view of the T cells’ secretory potential rather than a complete profile. In addition, the technology does not evaluate T-cell proliferation, an important functional metric. Moreover, IsoPlexis focuses on secretome activity, which does not equate directly to T-cell functionality—this is more accurately gauged by coculturing T cells with AML cells. These considerations are crucial when interpreting the outcomes of studies utilizing this technology.

Overall, this study represents a novel analysis comparing PB and BM compartments in patients with RelRef AML using pseudobulk and single-cell approaches, which has not been explored previously. Our findings indicate that it is important to sample the BM TME given that T-cell polyfunctionality differs between the systemic and local microenvironments. Furthermore, we demonstrate that post-IO cells are not unique at the single-cell level and that an unbiased, unsupervised clustering revealed novel functional groups that are potential biomarkers of response. These insights provide valuable information for understanding the interplay between different compartments and the potential use of polyfunctional analysis in predicting treatment outcomes.

## Supplementary Material

Supplementary Figure 1 Exploratory analysis of baseline T cell cytokine dynamics in PB and BM.

Supplementary Figure 2 Baseline correlation analysis of age with individual cytokines and functional groups.

Supplementary Figure 3 Baseline T cell individual cytokine activity in PB and BM.

Supplementary Figure 4 Baseline and post-therapy T cell functional activity within PB and BM.

Supplementary Figure 5 UMAP visualization depicting batch-corrected Seurat defined clusters.

Supplementary Table 1 Supplementary Table 1. Clinical Characteristics.
