# Supplementary figures and images for: Single-Cell CD4 and CD8 T-Cell Secretome Profiling Reveals Temporal and Niche Differences in Acute Myeloid Leukemia Following Immune Checkpoint Blockade Therapy
Source: Cancer Res Commun. 2024 Mar 6;4(3):671–81. doi: 10.1158/2767-9764.CRC-23-0402 (PMC10916538; doi:10.1158/2767-9764.CRC-23-0402)

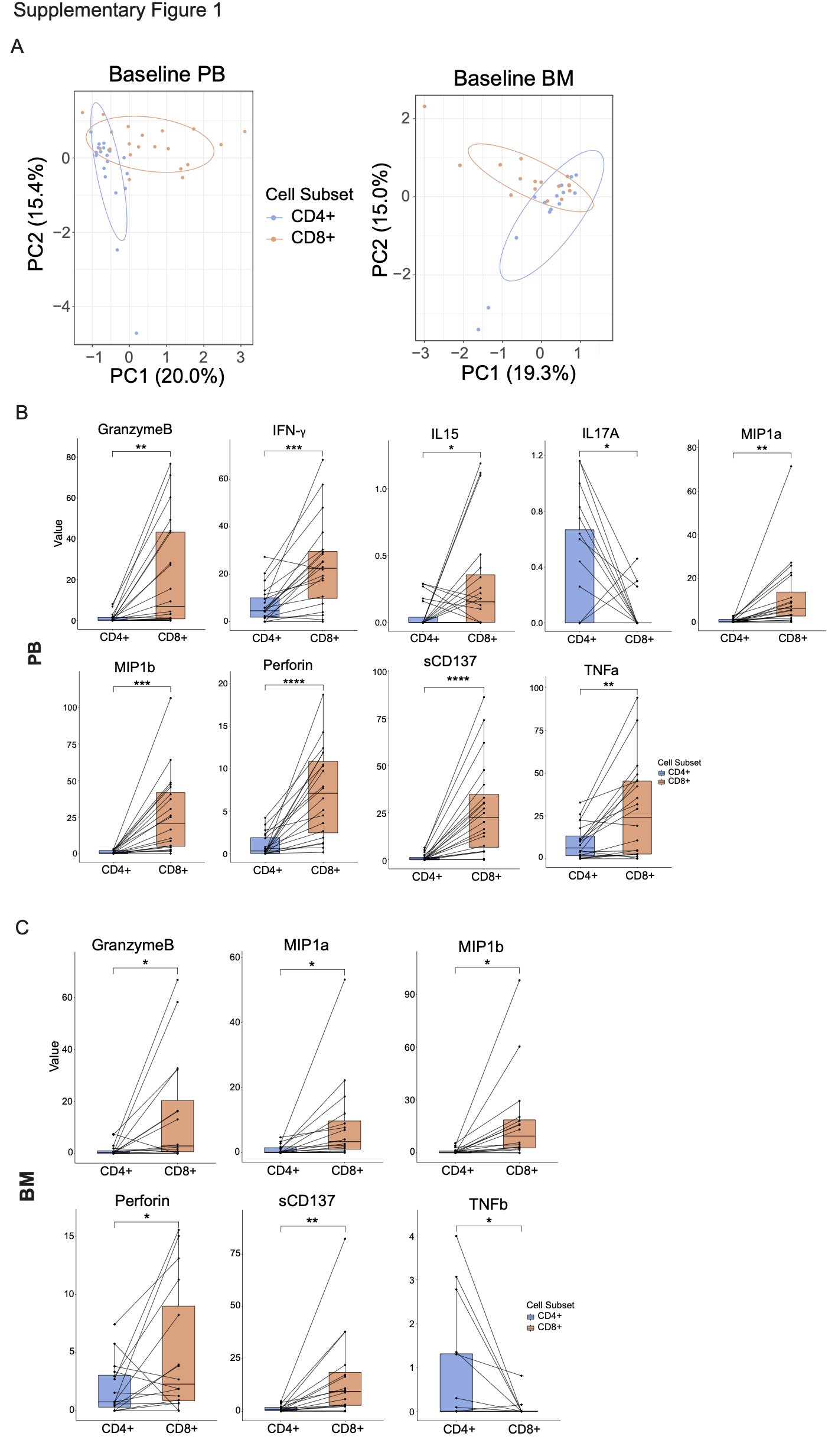

Supplement: Supplementary Figure 1 — Exploratory analysis of baseline T cell cytokine dynamics in PB and BM. [file crc-23-0402-s01.png]

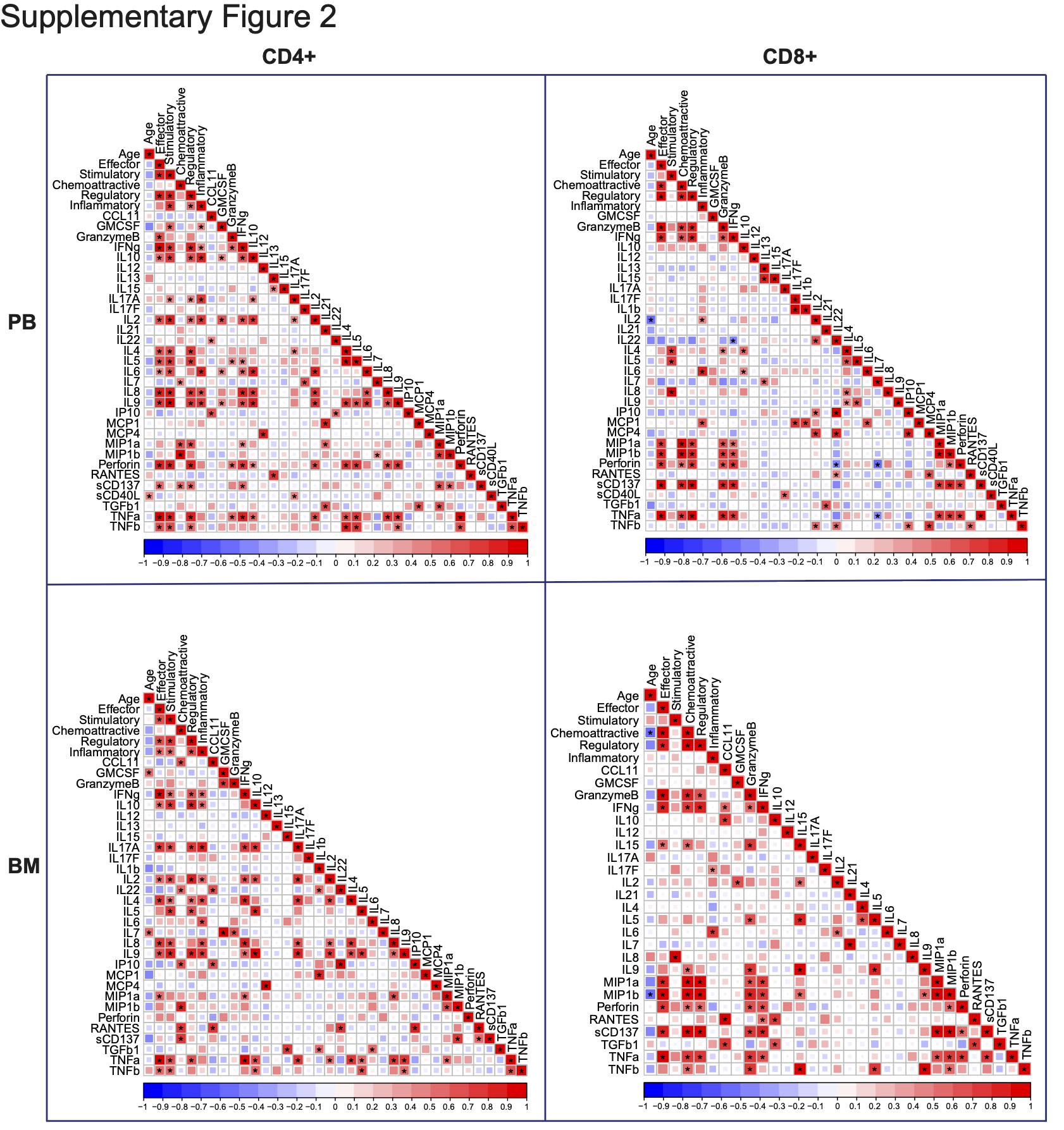

Supplement: Supplementary Figure 2 — Baseline correlation analysis of age with individual cytokines and functional groups. [file crc-23-0402-s02.png]

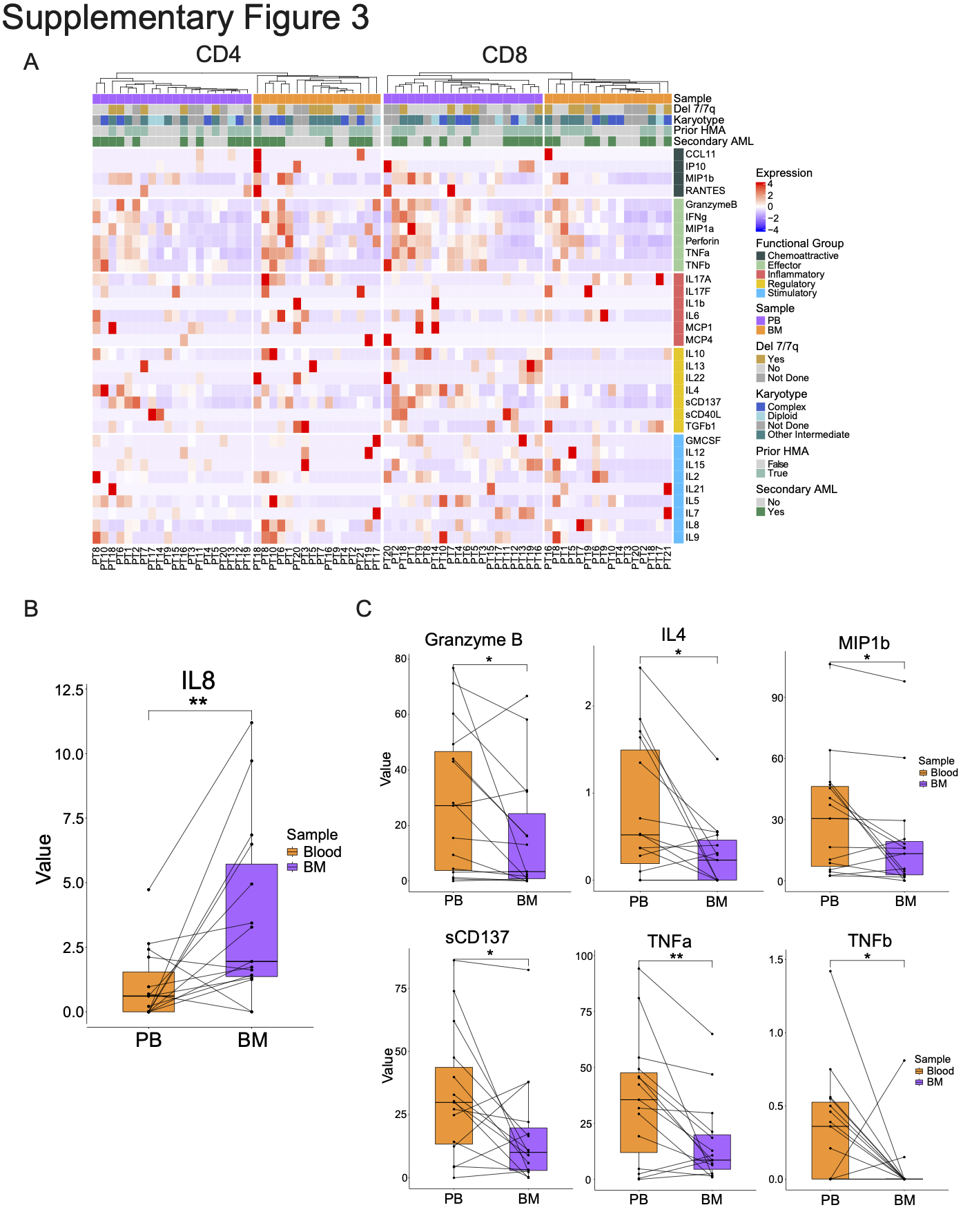

Supplement: Supplementary Figure 3 — Baseline T cell individual cytokine activity in PB and BM. [file crc-23-0402-s03.png]

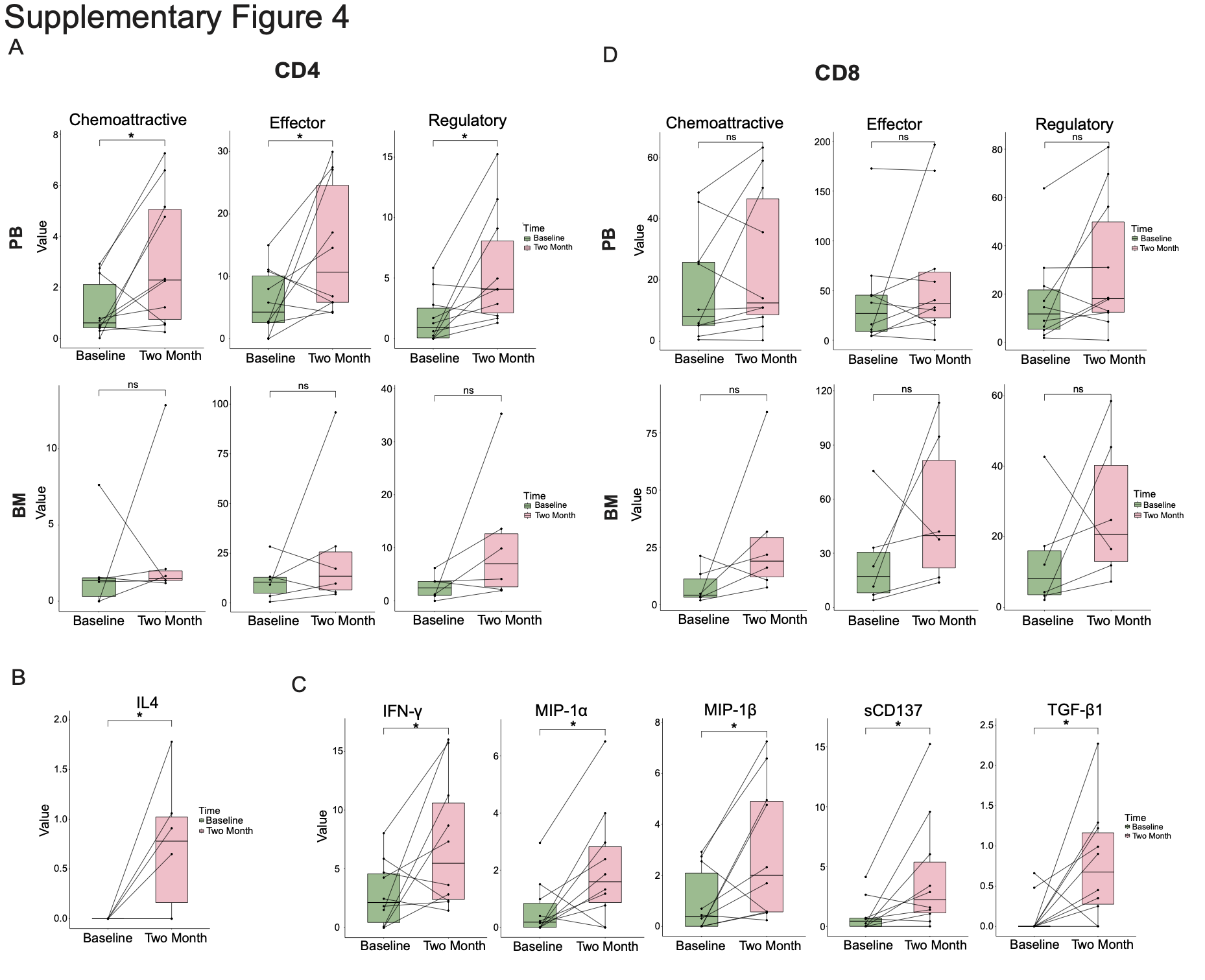

Supplement: Supplementary Figure 4 — Baseline and post-therapy T cell functional activity within PB and BM. [file crc-23-0402-s04.png]

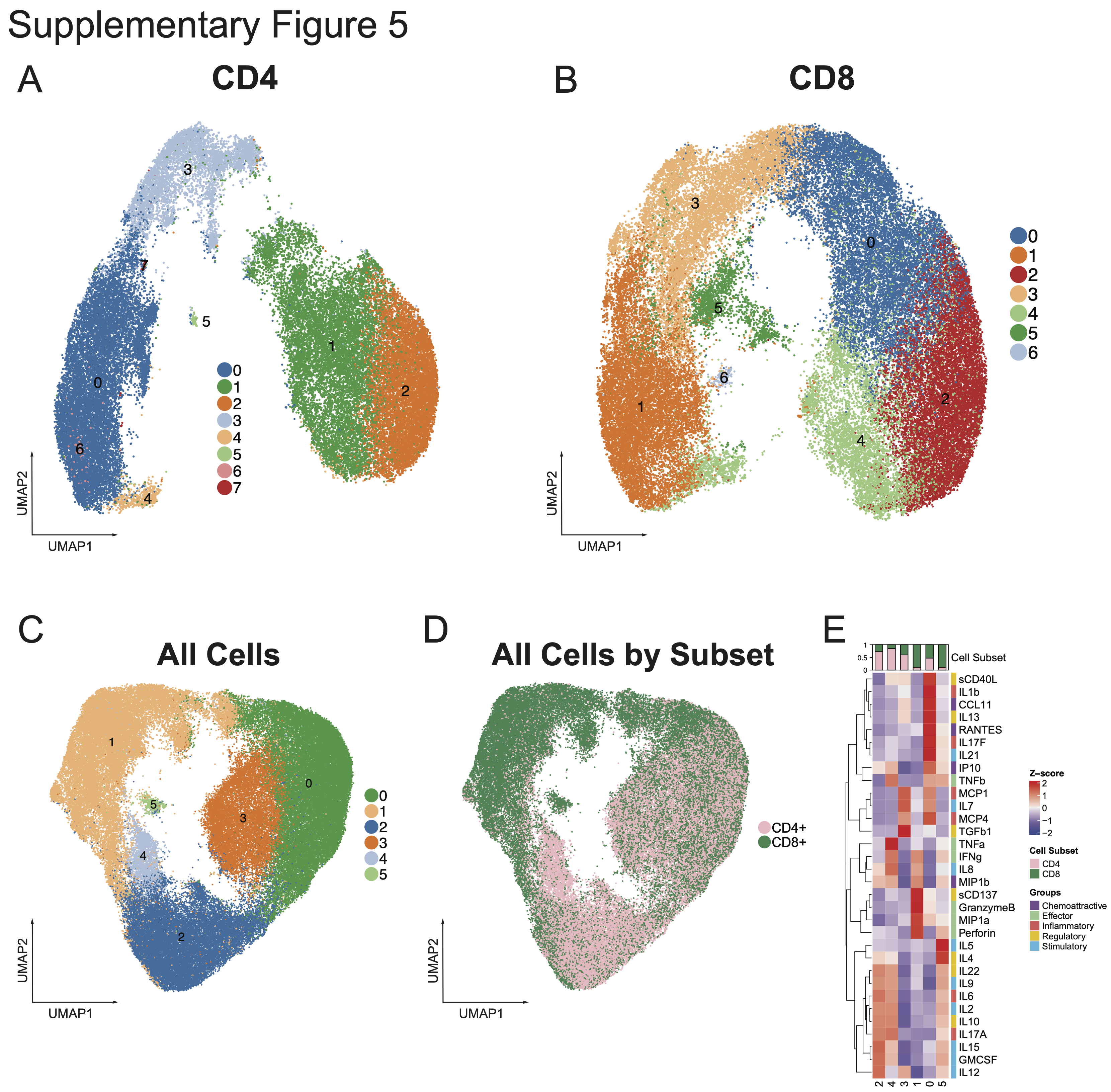

Supplement: Supplementary Figure 5 — UMAP visualization depicting batch-corrected Seurat defined clusters. [file crc-23-0402-s05.png]
