# Supplementary material for: Single-Cell CD4 and CD8 T-Cell Secretome Profiling Reveals Temporal and Niche Differences in Acute Myeloid Leukemia Following Immune Checkpoint Blockade Therapy
Source: Cancer Res Commun. 2024 Mar 6;4(3):671–81. doi: 10.1158/2767-9764.CRC-23-0402 (PMC10916538; doi:10.1158/2767-9764.CRC-23-0402)
Supplement: Supplementary Table 1 — Clinical Characteristics. [file crc-23-0402-s06.pdf]

Supplementary Table 1

Table 1. Clinical Characteristics

Characteristic	N = 21[1]
**Age**	47, 68, 90
**Gender**	
Female	6 (29%)
Male	15 (71%)
**Race**	
Black	2 (9.5%)
Latinx	2 (9.5%)
White	17 (81%)
**Acute Leukemia Subtype**	
AEL	1 (4.8%)
AML	19 (90%)
Megakaryocytic AML	1 (4.8%)
**Secondary AML**	
No	10 (48%)
Yes	11 (52%)
**Cytogenetic Group**	
Complex	5 (24%)
Diploid	3 (14%)
Other Intermediate	6 (29%)
Not Done	7 (33%)
**Prior HMA**	
No	10 (48%)
Yes	11 (52%)
**Prior SCT**	
No	17 (81%)
Yes	4 (19%)
**WBC**	0.6, 3.7, 49.6
**PB Blast %**	0, 11, 96
**Total Aza/Nivo Cycles**	2.0, 3.0, 27.0
**Treatment Response**	
CR/CRi/HI	10 (48%)
NR	11 (52%)
**Time to Response (months)**	0.85, 3.38, 12.65

[1]Minimum, Median, Maximum; n (%)
